# Nodular Regenerative Hyperplasia After Liver Transplant; It’s All in the Presentation

**DOI:** 10.3389/fsurg.2022.876818

**Published:** 2022-05-17

**Authors:** Allen K. Chen, Tyler Lunow-Luke, Seiji Yamaguchi, Claudia Praglin, Eliana Agudelo, Neil Mehta, Rachel Dirks, Hillary J. Braun, James M. Gardner, John P. Roberts, Shareef M. Syed, Garrett R. Roll

**Affiliations:** ^1^ Department of Surgery, Division of Transplant, University of California, San Francisco – Fresno, Fresno, San Francisco, CA; ^2^ Department of Surgery, Division of Transplant, University of California, San Francisco, San Francisco, CA; ^3^ Department of Medicine, Division of Gastroenterology, University of California, San Francisco, San Francisco, CA

**Keywords:** liver transplant, nodular regenerative hyperplasia (NRH), liver cancer, hepatology, liver disease, neoplasia, portal hypertension (PHTN)

## Abstract

There is a paucity of data on nodular regenerative hyperplasia after liver transplant. We aim to define the clinical disease trajectory and identify predictors of outcome for this rare diagnosis**.** This is a retrospective review of postulated risk factors and outcome in patients with nodular regenerative hyperplasia. Patients were classified as having a late presentation if nodular regenerative hyperplasia was diagnosed > 48 months from transplant, and symptomatic if portal hypertensive symptoms were present. Forty-nine of 3,711 (1.3%) adult recipients developed nodular regenerative hyperplasia, and mortality was 32.7% with an average follow up of 84.6 months. The MELD-Na 6 months after diagnosis did not change significantly. Patients with symptomatic portal hypertension at the time of diagnosis had a significantly higher risk of mortality (51.8%) compared to patients with liver test abnormalities alone (10.5%). 44.9% of patients had no previously postulated risk factor. Anastomotic vascular complications do not appear to be the etiology in most patients. The results suggest the vast majority of patients presenting with liver test abnormalities alone have stable disease and excellent long term survival, in contrast to the 56.3% mortality seen in patients that present more than 48 months after LT with symptomatic portal hypertension at diagnosis.

## Introduction

Nodular regenerative hyperplasia (NRH) of the liver is a rare condition that can occur before or after liver transplant (LT), characterized by transformation of liver parenchyma into hyperplastic parenchymal nodules. NRH is distinct from cirrhosis due to the absence of fibrosis (1). The etiology of NRH is idiopathic, but associations have been noted with collagen vascular, autoimmune, myeloproliferative and lymphoproliferative disorders, and medications including, chemotherapy and azathioprine (2–4). NRH can be an indication for LT. NRH after LT is exceedingly rare, the etiology is also unknown, and the prognosis is not well described (4). Patients with NRH after liver transplant can be asymptomatic with elevated aminotransferases and/or alkaline phosphatase, or can have symptoms from the sequelae of recurrent portal hypertension (variceal bleeding, ascites, hepatic encephalopathy, thrombocytopenia). Few cases of NRH after LT have been reported, and are mostly case reports and a small number of retrospective analyses (1, 4–6), the largest composed of 14 patients (4), leaving practitioners without a reliable road map.

The aim of this case series was to identify risk factors for the development of NRH after LT, define the disease trajectory, and identify predictors of progression of this disease.

## Patients and Methods

A retrospective chart review of patients in a prospectively collected database who underwent LT from 1988 to 2018 was performed. A database of patients with NRH after liver transplant has been maintained at the center. To ensure no patients with NRH were missed, we electronically queried all the biopsy reports from any patient who had a liver transplant for the words ‘nodular regenerative hyperplasia’ and then the authors reviewed those biopsies and added any patients not initially present in the database. These methods identified the final cohort of 49 patients who met the diagnostic criteria for NRH; histologic findings of benign transformation of hepatic parenchyma into small regenerative nodules without evidence of fibrosis. Patients for which the liver biopsy report listed NRH as a possible diagnosis were not included. Our center did protocol liver biopsies initially, but this practice stopped many years ago, so liver biopsy and ultrasounds for the vast majority of patients in this cohort were done for elevated liver tests, with or without symptoms of portal hypertension. Patient characteristics including age, sex, liver graft type, LT operative details, exposure to azathioprine and/or chemotherapy, liver allograft rejection episodes, vascular flow abnormalities seen on imaging within 30 days of the diagnosis of NRH, time from LT to diagnosis, total follow up time, symptoms, the need for evaluation for re-transplantation, MELD-Na score, allograft failure, and mortality were collected. Protocol liver biopsies were not done for a vast majority for the study period. Immune suppression was generally not altered as a result of the NRH diagnosis.

Patients were classified as having an early presentation (<48 months) versus a late presentation (>48 months), and as being asymptomatic at the time of diagnosis (biopsy done for liver test abnormalities) versus symptomatic (the presence of ascites, gastrointestinal bleed [GIB], hepatic encephalopathy, thrombocytopenia). Cumulative survival based on the clinical picture at the time of NRH diagnosis (early vs late, and symptomatic vs asymptomatic) was calculated, and a Kaplan-Meier analysis compared cumulative survival of the late symptomatic group versus all other groups. Statistical analyses were performed using the Statistical Package for Social Sciences (SPSS version 23.0, IBM). Multiple group comparisons were performed with Kruskal-Wallis and Chi square tests. Comparisons between two groups were done with Mann Whitney U and Fisher’s exact test. Significance was attributed to a *p* value <0.05. Continuous data are presented as mean ± standard deviation and categorical data are presented as percentages. The study was approved by the University of California, San Francisco Institutional Review Board (IRB# 18-26621).

## Results

During the study period 3,711 adult liver transplants were performed. Forty-nine patients (1.3%) developed NRH after LT. The etiology for LT in these patients is listed in Table 1. The mean age was 56.7 years at time of transplant, and 18 were female (36.7%). Seven patients (14.3%) were recipients of a living donor graft. The mean time from LT to diagnosis of NRH was 79.5 months (range 2–256 months), with an average follow up time from diagnosis of NRH of 84.6 months (range 2–254 months). Of existing postulated risk factors for NRH, 6 (12.2%) had a history of autoimmune conditions, 7 (14.3%) were exposed to azathioprine or 4 (8.2%) chemotherapy. Seventeen patients (34.7%) experienced at least one episode of rejection prior to diagnosis of NRH.

**Table 1:** Patient demographics.

*N*	49
Age (years)	56.7 ± 13.0
Male gender	31 (63.3%)
Etiology of liver failure	16 HCV 6 HBV 6 Cryptogenic Cirrhosis 5 EtOH 3 PBC 3 AIH 2 PSC 2 NASH 2 Fulminant 2 Biliary Atresia 1 TPN Cholestasis 1 Caroli’s
LDLT	7 (14.3%)
Time to diagnosis after transplant (mo)	79.5 ± 77.9
Vascular flow abnormalities on imaging	17 (34.7%)
Azathioprine exposure	7 (14.3%)
Chemotherapy exposure	4 (8.2%)
At least one rejection episode	17 (34.7%)
Autoimmune disease	6 (12.2%)
Time from NRH diagnosis to last follow up (mo)	84.6 ± 64.8
Time from NRH diagnosis to death or graft loss (mo)	45.4 ± 41.9
Deceased	18 (36.7%)
MELD-NA at diagnosis	12.0 ± 5.7
MELD-NA 6 mo after diagnosis	11.5 ± 4.7
T. Bili at diagnosis (mg/dL)	1.6 ± 1.9
T. Bili 6 mo after diagnosis (mg/dL)	1.9 ± 4.3
AST at diagnosis (U/L)	90.6 ± 153.4
AST 6 mo after diagnosis (U/L)	52.7 ± 44.9
ALT at diagnosis (U/L)	87.4 ± 100.4
ALT 6 mo after diagnosis (U/L)	52.8 ± 41.0
Alk Phos at diagnosis (U/L)	291.7 ± 221.9
Alk Phos 6 mo after diagnosis (U/L)	239.6 ± 185.9

*LDLT, living donor liver transplant; NRH, nodular regenerative hyperplasia; MELD-Na, model of end-stage liver disease score with serum sodium; T. Bili, total bilirubin; AST, aspartate aminotransferase; ALT, alanine aminotransferase; Alk Phos, alkaline phosphatase.*

**Figure 1:**
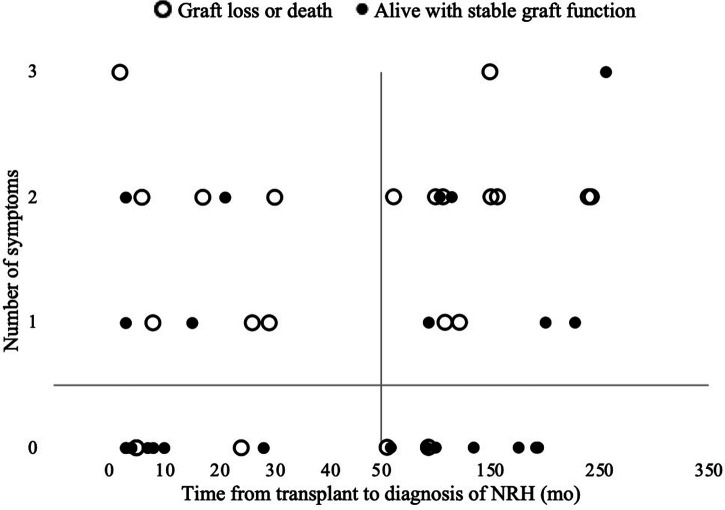
The of number of symptoms of portal hypertension present at diagnosis and time from LT to diagnosis of NRH.

The diagnosis of NRH did not appear to cluster at any time point of after transplant, rather it occurred at a relatively stable frequency over the follow up period (Figure 1 and data not shown). The liver test abnormalities did not point to hepatocyte injury or biliary injury per se, as generally there was a mild elevation in aspartate aminotransferase (AST), alanine aminotransferase (ALT) and a moderate elevation of alkaline phosphatase. The average MELD-Na score was 12.0 ± 5.7 at time of diagnosis, and 11.5 ± 4.7 at 6 months after diagnosis, suggesting the disease is not rapidly progressive. There were 18 deaths (36.7%) clustering in patients with a late symptomatic presentation, and death occurred an average of 49.1 months after diagnosis. The composite endpoint of death or graft failure occurred an average of 45.4 months after diagnosis.

**Figure 2:**
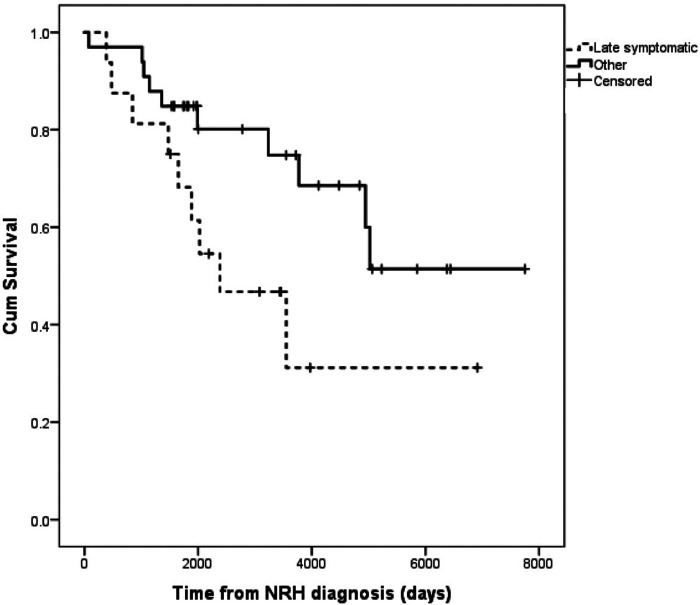
Kaplan-Meier survival of patients with late-symptomatic presentations of NRH versus all other patients with NRH.

The group that fared worst was the late symptomatic group, so this late symptomatic group (*n *= 16) was compared to all others (Table 2). This analysis identified no differences in age, gender, exposure to azathioprine, chemotherapy, number of rejection episodes, or the etiology of the initial chronic liver disease. All cause mortality over the follow up period was much more likely in late symptomatic subgroup (56.3% versus 27.3%, *p* = 0.071), and MELD-Na tended to be higher at the time of diagnosis (13.7 versus 11.1, *p* = 0.26)), and stayed significantly higher 6 months after diagnosis (14.6 versus 9.9, *p* = 0.01). The cumulative survival of the late-symptomatic group was worse than all other groups (Figure 2).

**Table 2:** Late symptomatic patients vs all others.

	Late symptomatic	All others	*p* value
*N*	16	33	–
Age (yrs)	59.1 ± 12.1	55.5 ± 13.4	0.39
Male gender	11 (68.8%)	20 (60.6%)	0.58
Time to diagnosis after transplant (mo)	152.4 ± 62.0	44.2 ± 57.9	<0.001
Vascular flow abnormalities on imaging	8 (50.0%)	9 (27.3%)	0.12
Azathioprine exposure	3 (18.8%)	4 (12.1%)	0.53
Chemotherapy exposure	2 (12.5%)	2 (6.1%)	0.44
Any rejection episodes	7 (43.8%)	10 (30.3%)	0.35
Number of rejection episodes	1.0 ± 0.6	0.6 ± 1.3	0.45
Autoimmune disease	2 (12.5%)	4 (12.1%)	0.97
MELD-NA at diagnosis	13.7 ± 6.7	11.1 ± 5.0	0.26
MELD-NA 6 months	14.6 ± 5.7	9.9 ± 3.1	0.010
LDLT	1 (6.3%)	6 (18.2%)	0.26
Graft failure	6 (37.5%)	5 (15.2%)	0.079
Reevaluated for transplant	6 (37.5%)	2 (6.1%)	0.005
Deceased	9 (56.3%)	9 (27.3%)	0.071

*MELD-Na, model of end-stage liver disease score with serum sodium; LDLT, living donor liver transplant.*

Vascular flow abnormalities were initially noted on abdominal Doppler ultrasound, and they involved the hepatic artery (*n* = 3), portal vein (*n* = 12), and inferior vena cava (*n* = 2). While 17 patients (34.7%) had vascular flow abnormalities identified on imaging, only 8 (16.3%) underwent intervention, suggesting the clinical team did not find them relevant in many cases, and most patients with NRH (83.7%) did not have a clinically relevant arterial or venous flow issue suggesting an anastomotic narrowing identified previously or around the time of diagnosis of NRH.

Of the NRH cohort, 14.3% of the patients received a graft from a living donor. Our center performed 265 adult living donor liver transplants over the study period, so 2.6% of all living donor recipients developed NRH, approximately double the frequency seen in our recipients of deceased donor allografts.

## Discussion

Nodular regenerative hyperplasia after LT is a rare finding on biopsy of unclear etiology and prognosis. The severely limited number of descriptions of this disease makes counseling patients with this finding on biopsy challenging. The incidence in our population was 1.3%. Anastomotic vascular complications after liver transplant are a principal postulated risk factor in patients who have NRH after transplant. We examined all imaging reports, operative notes, and invasive interventions undertaken in our cohort of 49 patients with NRH after LT and found that while 34.7% of patients had any abnormal finding on ultrasound around the time of NRH diagnosis only 16.3% required intervention for an anastomotic narrowing deemed to be relevant by the clinical team. This suggests that NRH can develop in the absence of an anastomotic vascular complication.

While no unifying risk factor was identified in our cohort, this data tends to direct attention towards an immune-mediated process. One issue that must be addressed when caring for patients with this diagnosis on biopsy after liver transplant is what to do with this immunosuppression. Our practice has been to leave immunosuppression unchanged. If the process is immune-mediated then one could postulate that increasing maintenance immunosuppression would be helpful. While that is possible, our data does not seem to support that. The excellent survival in patients with an early diagnosis suggests that it is reasonable to continue baseline immunosuppression rather than increasing the doses of these medications, as many of our patients lived with the disease for many years without progression.

The largest existing contemporary cohort of 14 patients with NRH after LT were grouped based on the length of time from LT to diagnosis and the presence or absence of symptomatic portal hypertension (4). This group reported the disease progressed even when it was first identified in patients that had no symptoms of portal hypertension. Our larger cohort with significantly longer follow up provides insight into the trajectory of this disease. We found that many with this diagnosis never progress to graft failure or death. Our highest risk group is patients that develop NRH more than 48 months after transplant and have symptomatic portal hypertension at the time of diagnosis. Importantly, all other patients, in fact most patients with NRH after liver transplant, have very good survival (Figure 2). The divergent findings between the two studies are possibly due to differences in study design/care algorithms, as those authors performed routine protocol ultrasounds (3 weeks after transplant) and liver biopsies (on 1 day, 4 months, and 1 year after transplant), which has not been our practice. Also, the mean follow up after liver transplantation presented here is longer (79.5 months versus 40.0 months, range 2–256 months versus 3–132 months, respectively). We suspect the poor survival in the group of patients with late symptomatic NRH is the result of multiple etiologies of chronic injury mounting in an ageing liver allograft, but this requires further study. Said another way, it is possible that NRH is a clinical entity that is identified in the background as more clinically impactful entities are being investigated, in which case NRH may not be driving the clinical outcome. This would require further study. It is interesting to note that time from transplant has more influence the risk of death or graft failure from NRH, and age at the time of LT does not.

Historically, vascular complications have been ascribed much importance in the development of NRH (4, 6). In our analysis, none of the risk factors for NRH (exposure to azathioprine and/or chemotherapy, autoimmune disease, or vascular flow abnormalities on imaging) were present in all patients with NRH, and none of these risk factors predicted graft loss or death. The slightly increased frequency of NRH in LDLT recipients is notable, and needs to be studied further given that autoimmune conditions such as primary biliary cirrhosis, primary sclerosing cholangitis and autoimmune hepatitis are somewhat more common in LDLT cohorts compared to patients undergoing deceased donor liver transplant.

We identified 2 patients with NRH that underwent a second LT. One patient was in the late symptomatic group who had a LT for HBV, but developed NRH 243 months after LT and had a second LT 22 years later. The second patient had the index LT for autoimmune hepatitis complicated by recurrence of autoimmune hepatitis and multiple episodes of rejection. She eventually underwent a second LT 16 years after first LT. NRH was only discovered on explant pathology. Her recovery after the second liver transplant was less eventful, but she was diagnosed with NRH 94 months after the second transplant when undergoing work up of elevated LFTs. She is the only patient we found that had NRH after LT twice. Neither of the patients described above was noted to have vascular flow abnormalities on imaging. Both patients are alive at the time of this analysis with functioning liver allografts.

Our study is the largest cohort of patients with NRH after LT, and has the longest follow up, but has shortcomings. This is a single center study, and is prone to the biases present in retrospective case series. It is possible that we failed to capture some patients with NRH, but we are relatively confident that our prospectively maintained database of NRH combined with the search of all liver biopsy results and explant pathology reports captured nearly all of the patients with this diagnosis. Also, the diagnosis of NRH can be a challenging clinical diagnosis to make, and low inter-observer agreement has been described (7). All the pathology specimens in this study were interpreted by specialized liver pathologists at a high-volume liver transplant center, but we do not have a control group. Given the rarity of this disease we would welcome a collaborative multicenter study effort.

In conclusion, this long-term retrospective study sheds light on this uncommon condition. The findings in our cohort are reassuring, as most patients with NRH after LT can be counseled the prognosis is quite good, the MELD after diagnosis is generally stable and disease progression is slow. In stark contrast, patients who present more than 48 months after LT and have symptomatic portal hypertension at the time of diagnosis have a 50% risk of mortality. The composite endpoint of death, or graft failure occurred an average of 45.4 months after diagnosis.

## Data Availability

The original contributions presented in the study are included in the article/supplementary materials, further inquiries can be directed to the corresponding author/s.
